# Rifampin Regulation of Drug Transporters Gene Expression and the Association of MicroRNAs in Human Hepatocytes

**DOI:** 10.3389/fphar.2016.00111

**Published:** 2016-04-26

**Authors:** Eric A. Benson, Michael T. Eadon, Zeruesenay Desta, Yunlong Liu, Hai Lin, Kimberly S. Burgess, Matthew W. Segar, Andrea Gaedigk, Todd C. Skaar

**Affiliations:** ^1^Division of Clinical Pharmacology, Department of Medicine, Indiana University School of MedicineIndianapolis, IN, USA; ^2^Department of Medical and Molecular Genetics, Indiana University School of MedicineIndianapolis, IN, USA; ^3^Department of Pharmacology and Toxicology, Indiana University School of MedicineIndianapolis, IN, USA; ^4^Division of Clinical Pharmacology, Toxicology and Therapeutic Innovation, Children's Mercy Kansas City and School of Medicine, University of Missouri-Kansas CityKansas City, MO, USA

**Keywords:** rifampin, drug transporter, gene expression, microRNA, human hepatocyte, ChIP-Seq, PXR binding site, RNA sequencing

## Abstract

Membrane drug transporters contribute to the disposition of many drugs. In human liver, drug transport is controlled by two main superfamilies of transporters, the solute carrier transporters (SLC) and the ATP Binding Cassette transporters (ABC). Altered expression of these transporters due to drug-drug interactions can contribute to differences in drug exposure and possibly effect. In this study, we determined the effect of rifampin on gene expression of hundreds of membrane transporters along with all clinically relevant drug transporters.

**Methods:** In this study, primary human hepatocytes (*n* = 7 donors) were cultured and treated for 24 h with rifampin and vehicle control. RNA was isolated from the hepatocytes, mRNA expression was measured by RNA-seq, and miRNA expression was analyzed by Taqman OpenArray. The effect of rifampin on the expression of selected transporters was also tested in kidney cell lines. The impact of rifampin on the expression of 410 transporter genes from 19 different transporter gene families was compared with vehicle control.

**Results:** Expression patterns of 12 clinically relevant drug transporter genes were changed by rifampin (FDR < 0.05). For example, the expressions of *ABCC2, ABCB1*, and *ABCC3* were increased 1.9-, 1.7-, and 1.2-fold, respectively. The effects of rifampin on four uptake drug transporters (*SLCO1B3, SLC47A1, SLC29A1, SLC22A9)* were negatively correlated with the rifampin effects on specific microRNA expression (SLCO1B3/miR-92a, SLC47A1/miR-95, SLC29A1/miR-30d#, and SLC22A9/miR-20; *r* < −0.79; *p* < 0.05). Seven hepatic drug transporter genes (*SLC22A1, SLC22A5, SLC15A1, SLC29A1, SLCO4C1, ABCC2, and ABCC4*), whose expression was altered by rifampin in hepatocytes, were also present in a renal proximal tubular cell line, but in renal cells rifampin did not alter their gene expression. *PXR* expression was very low in the kidney cells; this may explain why rifampin induces gene expression in a tissue-specific manner.

**Conclusion:** Rifampin alters the expression of many of the clinically relevant hepatic drug transporters, which may provide a rational basis for understanding rifampin-induced drug-drug interactions reported *in vivo*. The relevance of its effect on many other transporters remains to be studied.

## Introduction

The transport of drugs in and out of tissues and cells is now recognized as an important factor in drug efficacy and toxicity (International Transporter et al., 2010; Hillgren et al., 2013). Drug transport occurs at key sites including the liver, kidney, intestine, and blood-brain barrier to regulate drug and/or metabolite distribution (International Transporter et al., 2010; Hillgren et al., 2013). Understanding the regulation of drug transporter gene expression is important to improve drug efficacy and toxicity, especially for the many drugs transported into and out of the liver. Drug transport into and out of the liver is controlled by two superfamilies of transporters, the solute carrier transporters (SLC) and the ATP Binding Cassette transporters (ABC). Among these transporters, 32 have currently been designated as clinically relevant drug transporters (pharmADME.org; International Transporter Consortium; International Transporter et al., 2010; Hillgren et al., 2013). For most of these transporters, gene expression has not been studied in primary human hepatocytes to assess the impact of rifampin.

Rifampin is commonly used to treat tuberculosis worldwide. It is well-known to induce the expression of many of the cytochrome P450 drug metabolism genes. It is also known to regulate the gene expression of several *ABC* drug transporters, but its effects on many of the transporters is yet unknown (Stapelbroek et al., 2010; Peters et al., 2011; Vilas-Boas et al., 2013; Weiss and Haefeli, 2013). Several clinically significant rifampin-induced drug-drug interactions have been documented (Niemi et al., 2003; Baciewicz et al., 2013). At least some of the rifampin effect is due to its binding to and activation of the pregnane X receptor (PXR). PXR is a transcription factor that regulates the expression of several of the *ABC* transporter genes (Mottino, 2008). In addition, rifampin can also regulate transporter expression indirectly by regulating microRNAs (miRNAs), which target the transporter mRNAs (Haenisch et al., 2011). miRNAs are short RNA sequences that regulate mRNA function by degrading the target mRNAs or blocking the mRNA translation. Rifampin induces hepatic expression of many miRNAs (Ramamoorthy et al., 2013), and miRNAs have been shown to regulate drug transporter expression and translation (Haenisch et al., 2011; Zhu et al., 2011; Bang and Thum, 2012), supporting the possibility that miRNAs mediate some of the effects of rifampin on transporter expression. In fact, this has been shown to be true where rifampin induces miRNA-379 expression in the HepG2 human liver cancer cell line and this miRNA subsequently down-regulates ABCC2 protein levels (Haenisch et al., 2011).

In this report, we addressed three aims. First, we determined the effect of rifampin on the expression of the 32 clinically relevant drug transporter genes in primary human hepatocytes. Next, we determined the association of the rifampin-mediated changes in transporter expression with the rifampin-mediated induction of miRNA expression. Lastly, we evaluated whether the effects of rifampin on several drug transporters in hepatocytes has any tissue specificity. Our results demonstrate the wide-reaching effects of rifampin on the expression of many transporter genes relevant to drug metabolism and disposition.

## Methods

### Human hepatocytes, drug treatment, RNA isolation, and miRNA profiling

Our studies analyzed the RNA expression data collected by the studies done by Ramamoorthy et al. (2013). In those studies, “human hepatocytes from seven different subjects were obtained from CellzDirect (Durham, NC).” The primary human hepatocytes were grown on collagen coated plates and treated with rifampin (10 μM) or vehicle (methanol, 0.01%) for 24 h. Each culture from a different individual subject was treated as a biologic replicate (*n* = 7). *In vitro* studies were performed within 72–120 h from the time of hepatocyte isolation. Total RNA and miRNA were isolated with a miRNeasy kit (Qiagen, Valencia, CA). miRNAs (754) were analyzed using the Taqman OpenArray Human miRNA Panel in technical duplicates (each sample run on two OpenArrays). Global mRNA expression was measured by RNA-seq conducted on the SOLiD4 system (Life Technologies, Inc, Carlsbad, CA). The hepatocyte tissues were purchased commercially, and were de-identified. These studies were not deemed human subject research. CellzDirect was purchased by another company, and specific demographic or clinical information on the donors was not transferred in the transition and the information could not be retrieved.

### miRNA bioinformatics

The miRNA bioinformatic analysis was re-performed from the original analysis done by Ramamoorthy et al. (2013). For the technical duplicate values, the C_T_ values were averaged. If a miRNA had no value for both technical replicates, then it was assigned the highest C_T_ value of 40. In rare cases, if one technical duplicate detected the miRNA and the other technical duplicate was undetectable, the detectable value was used. To determine which miRNAs to include in the study, we selected only those with at least four of the seven control samples or four of the seven rifampin treated samples with average C_T_ values < 35. All the small nuclear RNA, U6 and RNUs, were removed. The remaining 333 miRNAs were Quantile normalized using PARTEK software. Quality control analysis after each normalization step included the visual evaluation of the histograms followed by principal component analysis (PCA) to identify systematic variations caused by various factors such as OpenArray runs. Normalized data were analyzed by ANOVA using a mixed effect model.

### RNA sequencing analysis

Our studies used the data processing, quality assessment, sequence alignment, and gene expression analysis that was previously performed and published by Ramamoorthy et al. (2013). As previously described for quality control, low quality. Briefly, RNA sequencing reads were removed including reads of less than 35 bases, or having a quality score of 20 or less. RNA was considered expressed if at least 10 reads were present after counts per million normalization.

Treatment and control groups were compared using the log_2_ transformed fold-change values. Of note, six data points in the transporter gene mRNA expression data were undetectable. Since a zero cannot be used in the log_2_ transformed fold-change calculations, the zero values were replaced with the minimum read numbers. The resultant *p*-values of the 410 transporters were corrected using a Benjamini and Hochberg's false discovery rate correction. A fold-change of one represents no change; a two-fold change is a doubling of the expression level.

Correlations of the rifampin effects on the expression of two transporter genes were performed using the Spearman rank correlations on the log_2_ transformed fold-change values. Correlations of the rifampin effect on specific gene expression with specific miRNA expression were also performed using the Spearman rank correlations on the log_2_ transformed fold-change using the delta C_T_ values for the miRNA expression.

### Selection of transporters

All human transporter genes were identified using the University of California Santa Cruz reference genome using the following inputs: http://genome.ucsc.edu/cgi-bin/hgTables, criteria- group: genes and gene prediction, track: RefSeq Genes, table: RefGene, assembly: feb. 2009 (GRch37/hg19), clade: mammal, genome: human, region: genome, output format: selected fields from primary and related tables, click get output button, then on the next screen, check both- Name [name of gen (usually transcript_id from GTF)], and name 2 [alternate name(e.g., gene_id from GTF)] and then click get output. Selected transporters evaluated in our study were queried from this UCSC gene list. These human transporters were selected using a combination of literature, pharmADME.org, and NIH NCBI Gene using the term transporter, and using terms transporter, influx transporter, efflux transporter. Transporter gene families were chosen based on possible relevance to drug metabolism or clinical relevance.

### Culture and rifampin treatment of renal cells

Normal human proximal tubular kidney (NHPTK) cells were a gift from the Robert L. Bacallao Laboratory (Herbert et al., 2013). NHPTK cells are primary renal cells from a single donor immortalized with human telomerase plasmid transfection and maintained in REGM media (Lonza, Basel, Switzerland) supplemented with 10% fetal bovine serum (HyClone) to maintain their renal phenotype. NHPTK cells were diluted to 20–30% confluence three times a week and maintained at 37°C in 95% humidified atmosphere with 5% CO_2_. All *in vitro* studies were performed on cells in passages 6 through 9 (corresponds to passage 3 through 6 following immortalization). Cultures from each passage were treated as biologic replicates (*n* = 4). NHPTK cells were treated for 24 h with rifampin (10 μM) or with the vehicle control of methanol (0.01%).

### Quantitative real-time PCR of renal cells

Quantitative real-time polymerase chain reaction (qRT-PCR) was performed to measure the level of expression of *CYP3A4, GAPDH*, and other transporter genes in NHPTK cells. Since, the PXR expression was at the limit of detection in NHPTK cells, we measured PXR in HepG2 cells as a positive control to verify that are assay could detect the low quantity of PXR found in HepG2 cells. A total of 1 million cells were pelleted following rifampin or vehicle treatment, washed in ice-cold PBS, centrifuged to remove PBS, and stored at −80°C until RNA isolation. Total RNA was extracted using the miRNeasy Plus Mini Kit (Qiagen, Hilden, Germany) following the manufacturer's protocol. RNA quality assessment and quantification were conducted using the optical spectrometry 260/280 and 260/230 nm ratios. Subsequently, mRNA was reverse transcribed to cDNA using the Bio-Rad iScript Reverse Transcription Kit (Bio-Rad, Hercules, CA). The final concentration of cDNA was 25 ng/ml. qRT-PCR was performed with TaqMan Gene Expression Assays (Bio-Rad) for *CYP3A4*, using commercially obtained primers on the Bio-Rad iCycler RT-PCR system as previously described (Eadon et al., 2013). qRT-PCR for all other genes, including *GAPDH* as an endogenous control, was performed using custom made primers (Life Technologies) and iTaq Universal SYBR Green (Bio-Rad) on the Bio-Rad iCycler RT-PCR system. Primer sequences are provided in Supplemental Table 1. Reactions were carried out in 20 μl volumes, which consisted of: 10 μl SYBR green, 4 μl cDNA, 0.4 μl of each primer (10 μM stock), and 5.2 μl of water. The RT-PCR reactions were run in triplicate. The thermocycler parameters were 95°C for 30 s, 40 cycles of 95°C for 15 s and then an annealing temperature for 30 s (provided in Supplemental Table 1), with ramping speeds of 1.6–1.98 degrees °C/s and a melt curve. The C_T_ threshold and baseline for each experiment were set automatically by the Bio-Rad software. The delta-delta (ΔΔC_T_) method was used to obtain the relative expression of each gene for samples treated with rifampin or vehicle. Each sample's gene expression of interest was first subtracted from its *GAPDH* expression to determine its ΔC_T_. The ΔC_Tcontrol_ was then subtracted from the ΔC_Trifampin_ to determine the ΔΔC_T_. Fold-changes of the rifampin treatments as compared to the controls were determined by the formula: fold change = 2^ΔΔCT^. Gene expression for each condition is given as a fold-change ± SEM relative to the control where the SEM was calculated from individual ΔΔC_T_s of the 4 biologic replicates for each condition. Statistical significance was assessed based on a student's *t*-test between conditions.

### *In silico* ChIP-Seq PXR binding site analysis

A publically available chromosome immunoprecipitation DNA sequencing (ChIP-Seq) database available from Smith et al. (2014) was analyzed *in silico* for PXR binding sites within the 410 transporter genes in this study (Smith et al., 2014). This ChIP-Seq dataset analyzed the PXR binding in rifampin treated vs. DMSO vehicle treated HepG2 cells.

### Accession numbers

The raw RNA-seq data have been made publically available through NCBI's Gene Expression Omnibus and are accessible through GEO Series accession number GSE79933 (http://www.ncbi.nlm.nih.gov/geo/query/acc.cgi?acc=GSE79933). The miRNA OpenArray data have been made publically available at http://compbio.iupui.edu/group/6/pages/rifampin.

## Results

### Regulation of membrane transporters by rifampin

A total of 410 transporter genes from 19 transporter gene families were selected for analysis in the primary hepatocytes. Two hundred sixty-seven of the genes were in the *ABC* and *SLC* superfamilies of transporters. Thirty-four of the 267 are drug transporters that are considered to be clinically important for drug disposition (International Drug Transporter Consortium and/or PharmADME.org; International Transporter et al., 2010; Hillgren et al., 2013). Of the 32 transporters designated as “clinically relevant drug transporters” by the International Transporter Consortium (Table 1), we expected at least 18 to be expressed in the hepatocytes (Hillgren et al., 2013). All 18 expected drug transporter genes were indeed found to be expressed in every donor sample; we also found six additional transporters that were previously not reported to be expressed in the liver (Table 2). The expression of 12 of these drug transporter genes was modulated in the hepatocytes by rifampin (Table 2). Rifampin induced the expression of 9 of the 12 drug transporter genes and the expression of three transporters was reduced. The results of the other 16 families of transporters (143 genes) we have characterized are provided in Supplemental Table 2.

**Table 1:** **Clinically important drug transporters**.

**Influx**	**Gene name**	**Efflux**	**Gene name**	**Influx/Efflux**	**Gene name**
^*^MCT1	**SLC16A1**	^*^MDR1	**ABCB1**	^*^ENT1	**SLC29A1**
^*^PEPT1	**SLC15A1**	^*^BCRP	**ABCG2**	^*^ENT2	**SLC29A2**
^*^OCT1	**SLC22A1**	^*^MRP2	**ABCC2**	^*^OSTalpha	**SLC51A**
^*^OAT2	**SLC22A7**	^*^MRP3	**ABCC3**	^*^OSTbeta	**SLC51B**
^*^OATP4C1	**SLCO4C1**	^*^MATE1	**SLC47A1**	^*^OCTN2	**SLC22A5**
^*^OATP2B1	**SLCO2B1**	^*^MRP4	**ABCC4**	----------------------------	
^*^NTCP	**SLC10A1**	^*^MRP6	**ABCC6**	OAT4	SLC22A11
^*^OATP1B3	**SLCO1B3**	^*^BSEP	**ABCB11**	OCTN1	SLC22A4
^*^OATP1B1	**SLCO1B1**	^*^MRP5	**ABCC5**		
^*^OAT7	**SLC22A9**	-----------------			
-----------------		MATE2-K	SLC47A2		
ASBT	SLC10A2				
PEPT2	SLC15A2				
URAT1	SLC22A12				
OAT3	SLC22A8				
OAT1	SLC22A6				
OCT2	SLC22A2				
OATP1A2	SLCO1A2				

Clinical importance of drug transporters as determined by the International Transporter Consortium, and pharmADME.org.

* *(**Bold**) The 24 genes with detectable expression in the primary human hepatocytes*.

**Table 2:** **Rifampin-induced changes in gene expression of clinically relevant drug transporters**.

**Common name**	**Gene name**	**Fold change**	***p*-value**	**FDR**	Example of substrates^*^
OSTbeta	*SLC51B*	12.67	1.20E-113	4.90E-111	Bile acids, conjugated steroids
MRP2	*ABCC2*	1.85	3.56E-23	2.92E-21	Chemotherapy, statin, antepileptics, tamoxifen, DMARDs, antidepressants, clopidogrel, anti-retroviral
MDR1	*ABCB1*	1.72	6.63E-18	3.02E-16	Chemotherapy, statin, antepileptics, tamoxifen, DMARDs, antidepressants, clopidogrel, anti-retroviral
OATP2B1	*SLCO2B1*	1.73	2.34E-16	8.73E-15	Fexofenadine, montelukast, grapefruit, orange juice, apple juice, endogenous steroids
MRP6	*ABCC6*	1.65	5.82E-14	1.84E-12	Thalidomide, docetaxel
OATP1B3	*SLCO1B3*	−1.57	2.15E-11	5.18E-10	Irinotecan, docetaxe, myophenolate, statin, methotrexate
OATP4C1	*SLCO4C1*	1.46	2.84E-07	3.88E-06	Estrone 3-sulfate
MATE1	*SLC47A1*	1.37	2.64E-06	2.84E-05	Meformin, trimethoprim
ENT1	*SLC29A1*	1.38	4.68E-06	4.80E-05	Gemcitabine, paclitaxe, gemcitabine, peginterferon, ribavirin
OAT7	*SLC22A9*	−1.34	9.76E-05	0.0007	Methotrexate
PEPT1	*SLC15A1*	−1.27	5.01E-04	0.0031	Statin, oseltamivir
MRP3	*ABCC3*	1.18	0.0098	0.038	Cisplatin, MTX, doxorbuicn, cyclophosphamide, clopidogrel, codiene, morphine, etoposide
OSTalpha	*SLC51A*	1.21	0.014	0.050	Bile acids, conjugated steroids
BCRP	*ABCG2*	1.18	0.015	0.054	Antiepileptics, MTX, statin, anti-retrovirals, tecans, anti-neoplastics (irinotecan, platinum, anthracycline, taxane) acetaminophen, uric acid
OCT1	*SLC22A1*	−1.16	0.019	0.060	Morphine, metformin, tramadol, antiemetics, dopamine agonist, antiretrovirals, sorafinib
OAT2	*SLC22A7*	−1.15	0.040	0.11	Fluoropyrimidine, zidovudine
MRP5	*ABCC5*	1.17	0.066	0.16	Antiepileptics, irinotecan
MRP4	*ABCC4*	1.17	0.090	0.21	cisplatin, antiretroviral, bisphosphonates, MTX, acetaminophen, azathioprine, mercaptopurine
NTCP	*SLC10A1*	−1.08	0.21	0.39	Bile acids
OCTN2	*SLC22A5*	−1.08	0.30	0.51	Imatinib, oxaliplatin
BSEP	*ABCB11*	−1.07	0.32	0.55	Statin
OATP1B1	*SLCO1B1*	1.04	0.57	0.74	Simvastatin, statin, rifampin, irinotecan, methotrexate, thyroid, estrone sulfate, angiotensin receptor blocker, MTX, caspofungin, ace inhibitor
MCT1	*SLC16A1*	1.03	0.61	0.78	Lactate
ENT2	*SLC29A2*	−1.03	0.89	0.94	Gemcitabine, thiopurine

Primary human hepatocytes were treated with 10 μM Rifampin or vehicle for 24 h. Fold-change is the ratio of gene expression treatment/control. A fold-change equal 1 is defined as no change, and a fold change of 2 is defined as 2x the expression. FDR, False Discovery Rate.

* *Cited from PharmGKB.org*.

**Figure 1:**
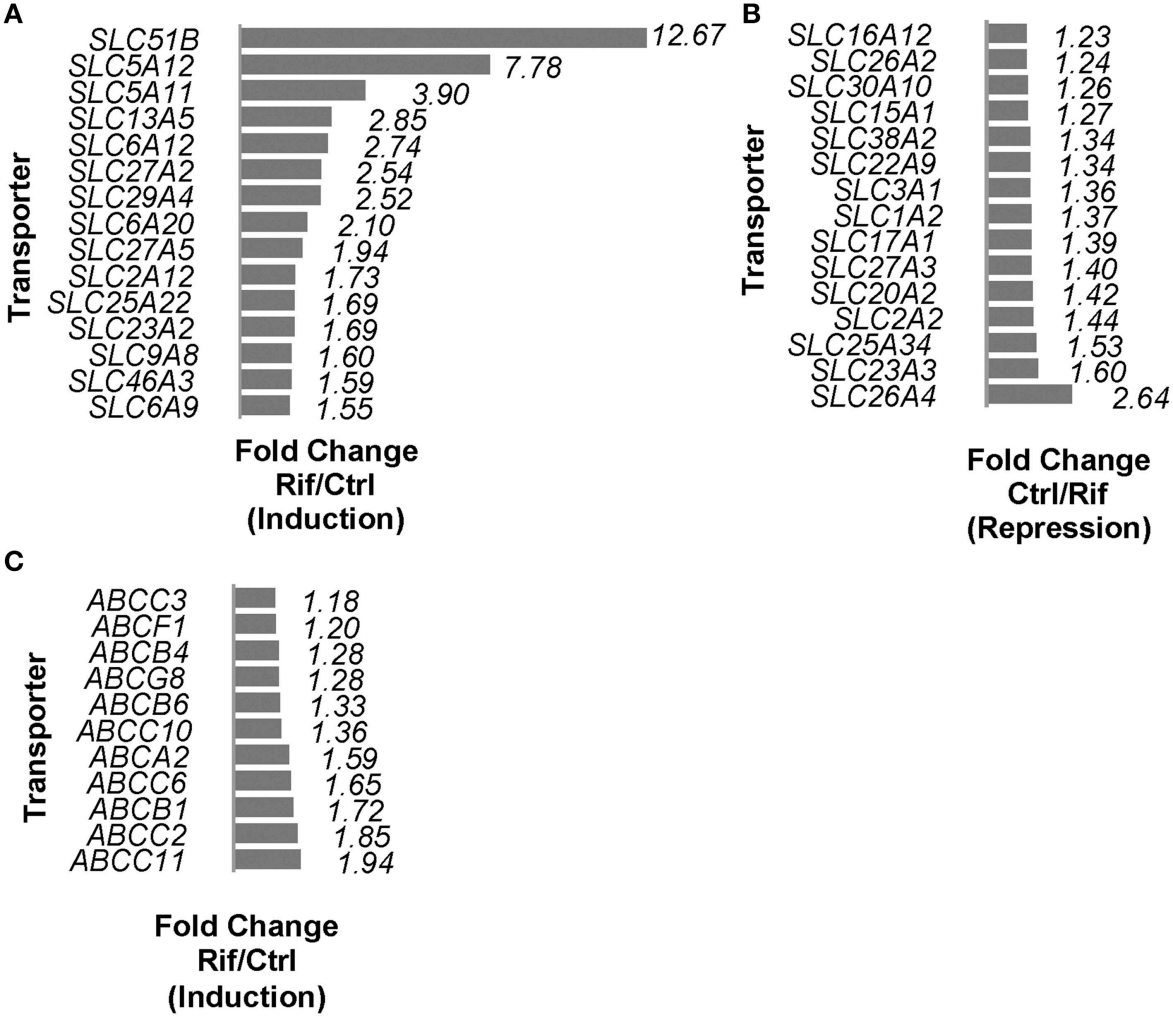
*****SLCs*** (including ***SLCOs***) and ***ABCs*** mRNA expressions regulated by rifampin with FDR < 0.05. (A)** Induction of *SLCs* by rifampin by fold change (fold change represented as rifampin/control) (FDR < 0.05); the top 15 *SLCs* are shown. **(B)** Reduction of *SLCs* by rifampin by fold change (fold change represented as control/rifampin) (FDR < 0.05); the 15 most reduced *SLCs* are shown. **(C)** Rifampin induced 11 *ABC* transporter genes based on fold change (rifampin/control) with FDR < 0.05. A fold change equal to 1 is defined as no change, and a fold change of 2 is defined as 2x the expression.

Rifampin altered the expression of 62 of the 267 (~23%) solute carrier (*SLC*) superfamily genes (Supplemental Table 2). The fifteen *SLC* genes with the highest changes in expression levels are shown in Figures 1A,B. The expression of three out of the five (60%) detected genes of the *SLCO* family were also altered by rifampin (Supplemental Table 2). PXR has been shown to regulate *SLCO2B1* and *SLCO1B1*, but the expression of only *SLCO2B1* increased (1.73-fold) in our study. In addition, rifampin altered the expression of 12 of the 38 (32%) ATP-binding cassette (*ABC*) superfamily transporter genes (Supplemental Table 3). Eleven of these *ABC* genes were induced as shown in Figure 1C, and one (*ABCA8*) was reduced (Supplemental Table 2).

### Correlations of the effect of rifampin on gene expression

**Figure 2:**
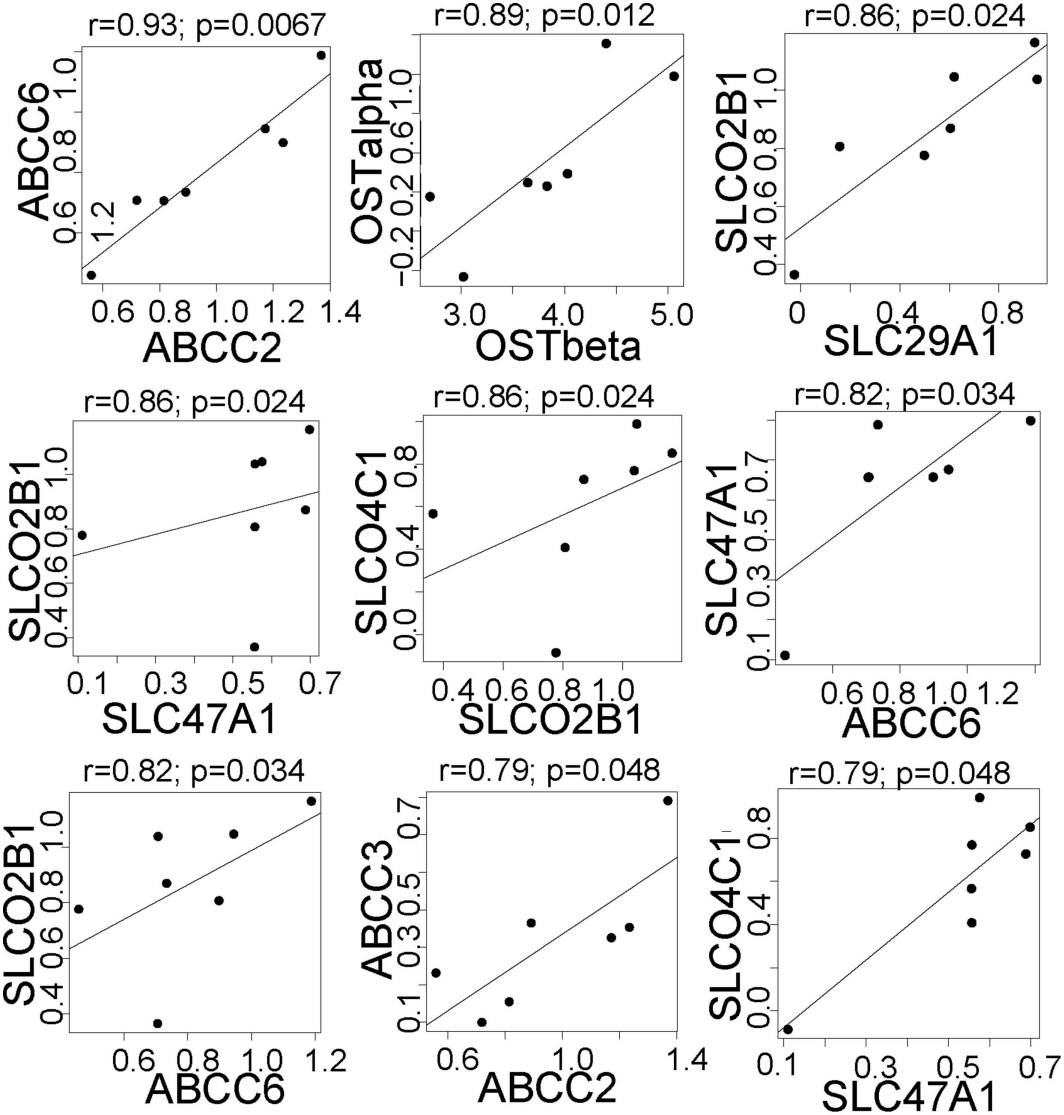
**The significant correlations between the expression of clinically relevant transporter genes that were regulated by rifampin**. The expression [represented by log_2_fold change (Log_2_FC)] after rifampin treatment) was correlated for all 410 transporter genes. Only the significant correlation (*p* < 0.05 by Spearman correlation) are shown for transporters regulated by rifampin with an FDR < 0.05. Spearman correlation was performed because an assumption of normal distribution could not be made.

**Figure 3:**
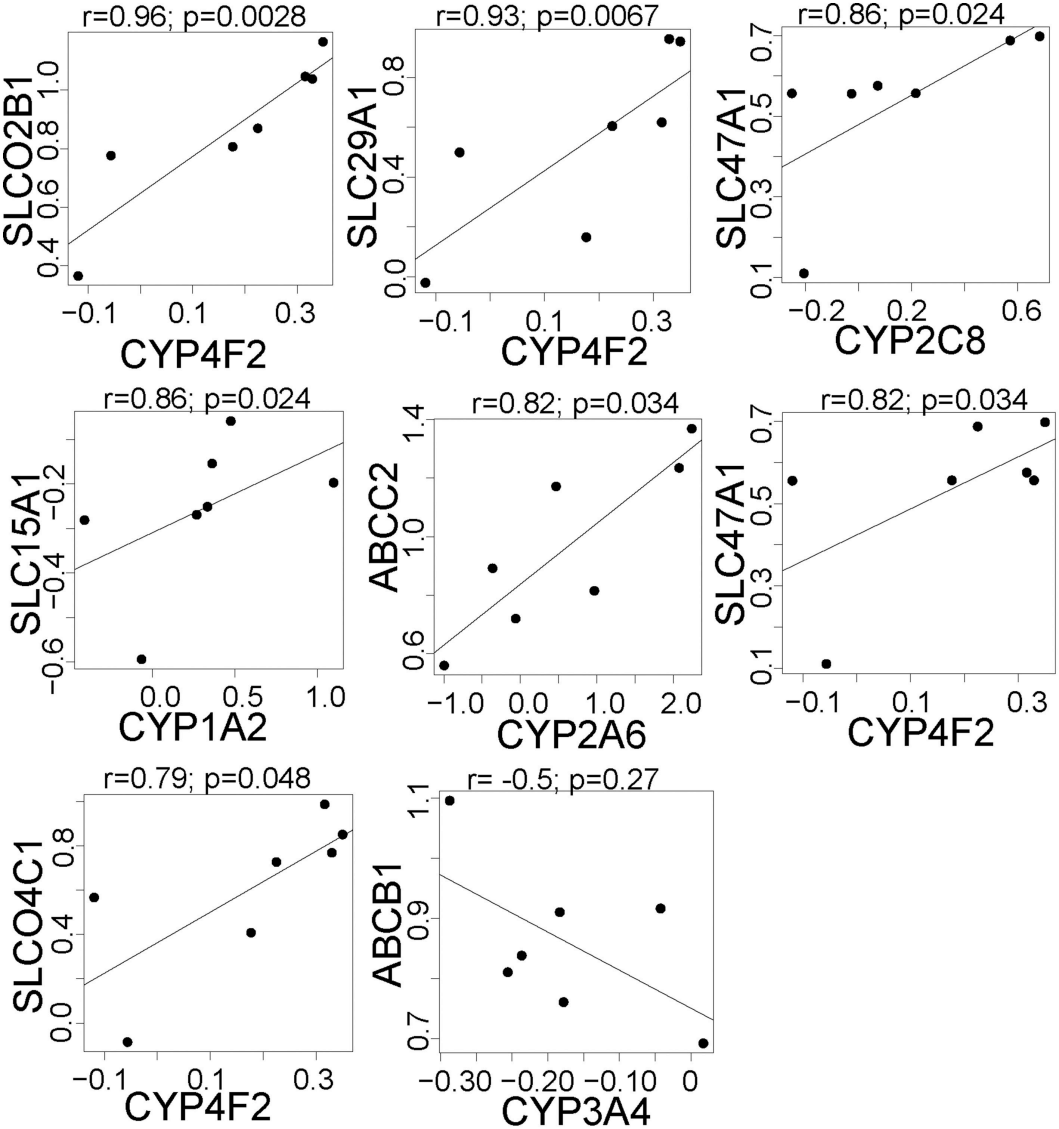
**The significant correlations between the expression of drug metabolism genes and clinically relevant transporter genes that are regulated by rifampin**. The expression [represented by log2fold change (Log2FC)] after rifampin treatment was correlated between all CYP and 410 transporters genes. The figure illustrates, the seven significant positive and one significant negative correlations between the expression of CYP and drug transporter genes that were regulated by rifampin with an FDR < 0.05. These were the only correlations with a *p* < 0.05 by Spearman correlation. Spearman correlation was performed because an assumption of normal distribution could not be made.

We next tested whether there were correlations in the effect of rifampin on the expression of drug transporter and cytochrome P450 (*CYP)* genes. Since each hepatocyte culture replicate was from a different donor, we considered each replicate a separate individual. There were nine pairs of drug transporters that were positively correlated (FDR < 0.05; Figure 2; all CYP/Transporter gene expression correlations are found in Supplemental Table 3), i.e., each pair of genes was upregulated (*r* > 0.79) upon rifampin treatment. We also correlated the rifampin effects on the drug transporters with rifampin effects on *CYP* gene expression. There were seven positive correlations (FDR < 0.05; Figure 3; for the correlations of all CYP/Transporter genes see Supplemental Table 4). Interestingly, four were correlations with *CYP4F2* (*SLCO2B1, SLC29A1, SLC47A1*, and *SLCO4C1*).

### Correlation between miRNA induction and changes of clinically relevant drug transporter expression

We have previously shown that rifampin induces the expression of several miRNAs in hepatocytes (Ramamoorthy et al., 2013). To identify drug transporters that may be modulated by these miRNAs, we determined the correlations between rifampin induction of miRNAs and the rifampin effect on drug transporter gene expression. The direct effects of the miRNAs on gene expression would be expected to downregulate transporter expression; however, since miRNAs could also have indirect effects and induce the expression of the drug transporter genes, we searched for both positive and negative correlations. We identified 14 highly correlated negative associations (Table 3; for the correlations of the all transporter/miRNAs gene expression see Supplemental Table 5). Three miRNAs, miR-320, miR-616, and miR-95, each correlated with two different drug transporters. Three drug transporters, *ABCB11, ABCC4, and ABCC5* were negatively correlated with two different miRNAs each. Based on the bioinformatics tools we employed, none of these were predicted to be direct effects. Only three rifampin-induced miRNAs were predicted to bind to drug transporters. Target Scan predicted miRNA binding of miR-335 to *SLC29A2*, miR-200b# to *SLCO1B1*, and miR-616 to *SLCO1B1*. However, neither of these transporters genes expression were altered by rifampin.

**Table 3:** **All clinically relevant drug transporters correlated with miRNA induced by rifampin with a ***p*** < 0.05**.

**miRNA**	**Gene**	**Spearman correlation**	***p*-value**	**miRNA**	**Gene**	**Spearman correlation**	***p*-value**
**Positive correlation**				**Negative correlation**			
hsa-miR-200b#	*SLCO1B1*	0.96	0.0028	hsa-miR-107	*SLC16A1*	−0.96	0.0028
hsa-miR-766	*ABCG2*	0.96	0.0028	hsa-miR-202	***SLC22A9***	−0.93	0.0067
hsa-miR-660	*ABCB11*	0.96	0.0028	hsa-miR-616	*SLC22A5*	−0.93	0.0067
hsa-miR-660	*ABCC6*	0.93	0.0067	hsa-miR-95	*ABCB11*	−0.93	0.0067
hsa-miR-616	*ABCC5*	0.89	0.012	hsa-miR-335	*ABCC4*	−0.93	0.0067
hsa-miR-886-3p	***SLC51B***	0.89	0.012	hsa-miR-320	*ABCC5*	−0.89	0.012
hsa-miR-886-3p	*SLC51A*	0.89	0.012	hsa-miR-638	*ABCC5*	−0.86	0.024
hsa-miR-766	*SLCO1B1*	0.86	0.024	hsa-miR-186	*SLC29A2*	−0.86	0.024
hsa-miR-660	***SLC47A1***	0.86	0.024	hsa-miR-92a	*ABCC4*	−0.83	0.021
hsa-miR-335	*SLC29A2*	0.86	0.024	hsa-miR-320	*ABCB11*	−0.82	0.034
hsa-miR-107	*SLC22A7*	0.82	0.034	hsa-miR-616	*SLCO1B1*	−0.79	0.048
hsa-miR-660	***SLCO2B1***	0.82	0.034	hsa-miR-30d#	***SLC29A1***	−0.79	0.048
hsa-miR-660	***SLCO4C1***	0.82	0.034	hsa-miR-95	***SLC47A1***	−0.79	0.048
hsa-let-7g	*SLC51A*	0.82	0.034	hsa-miR-92a	***SLCO1B3***	−0.76	0.049
hsa-miR-766	*SLCO2B1*	0.79	0.048				
hsa-miR-660	***ABCC2***	0.79	0.048				
hsa-miR-660	*SLC22A1*	0.79	0.048				

*Correlation was assessed for all miRNA tested and the 410 transporters expressed in hepatocytes. Shown in this table are the all the clinically relevant drug transporters regardless of rifampin regulation with the miRNAs induced by rifampin with a p < 0.05. In bold, the subset of the only clinically relevant transporters whose expression was regulated by rifampin with an FDR < 0.05 and correlated with rifampin induced miRNAs. Spearman correlation selected as samples because assumption of normal distribution could not be made*.

We identified 18 highly positive correlations between the rifampin effects on miRNAs and its effects on drug transporter expression levels (Table 3; Supplemental Table 5). miR-660 positively correlated with seven different transporters, miR-766 positively correlated with three transporters, and miR-886-3p and let-7g positively correlated with two transporters. None of the rifampin-induced miRNAs were predicted to bind to any of the rifampin-regulated drug transporters.

### Rifampin's modulation of drug transporter gene expression is tissue specific

Many of the drug transporters that we detected in the hepatocytes are also expressed in other tissues, including the kidney (Hillgren et al., 2013). Thus, we determined if rifampin demonstrated any tissue specificity by regulating the same transporters in human kidney cells. Normal human proximal tubular kidney (NHPTK) cells were treated with rifampin analogously to primary hepatocytes, and gene expression was measured. Seven transporters were selected based on their differential expression in hepatocytes and their renal proximal tubular relevance. Expression of *SLC22A1, SLC22A5, SLC15A1, SLC29A1, SLCO4C1, MRP2, and MRP4* was detected, but did not change significantly following rifampin treatment (Supplemental Figure 1A; statistics are provided in Supplemental Table 6). *CYP3A4* expression was low in NHPTK cells with raw C_T_ values between 35 and 37. Expression was not significantly elevated following rifampin treatment (Supplemental Figure 1B). As CYP3A4 is PXR dependent, gene expression of *NR1I2 (PXR)* was also measured in NHPTK cells and compared to *NR1I2* expression in a positive control cell line, HepG2 cells. Since we expected *NR1I2* expression to be much lower than hepatocytes, we used HepG2 as a positive control, which is known to have relatively low *NR1I2* expression levels (Gerets et al., 2012). The expression of *NR1I2* was much lower in NHPTK cells when compared to HepG2 (Supplemental Figure 1C).

### 38 Transporters have rifampin induced PXR binding sites

Utilizing a publically available ChIP-Seq database from Smith et al. (2014), Figure 4 illustrates our *in silico* analysis of this dataset. The 410 transporter genes were mapped to Ensembl annotation. The transcription start site (TSS) was located for each gene and a promoter region was defined as ± 2 kilobases from TSS of each gene. From the ChIP-Seq dataset, 6302 PXR peaks were identified in the rifampin treated HepG2 cells and 1157 PXR peaks in DMSO treated HepG2. PXR peaks were extracted only if uniquely found in rifampin treated HepG2 vs. DMSO vehicle. The remaining 6063 unique rifampin peaks were compared for overlap with the 410 promoter regions of the transporters. 58 of the 410 transporter genes' promoter regions were identified to have PXR binding sites. Table 4 illustrates the PXR binding site for one isoform of each gene transcript, while Supplemental Table 7 provides a list of the PXR binding site analysis for all gene transcript isoforms. Thirty three members of the *SLC* (*SLC10A7, SLC16A3, SLC17A5, SLC1A4, SLC20A1, SLC20A2, SLC22A15, SLC22A3, SLC23A2, SLC25A1, SLC25A10, SLC25A11, SLC25A22, SLC25A4, SLC25A42, SLC25A5, SLC26A2, SLC29A1, SLC2A1, SLC2A10, SLC30A10, SLC37A3, SLC39A7, SLC39A8, SLC3A2, SLC41A2, SLC43A1, SLC47A1, SLC5A11, SLC5A3, SLC5A9, SLC7A5*, and *SLCO2B1*), and 7 members of the *ABC* (*ABCA2, ABCA3, ABCA9, ABCB4, ABCC10, ABCC2*, and *ABCC3*) superfamily of transporters have PXR binding sites. Five of the clinically relevant drug transporter genes, *ABCC2, ABCC3, SLC29A1, SLC47A1*, and *SLCO2B1* were found to have PXR peaks within their promoter regions.

**Figure 4:**
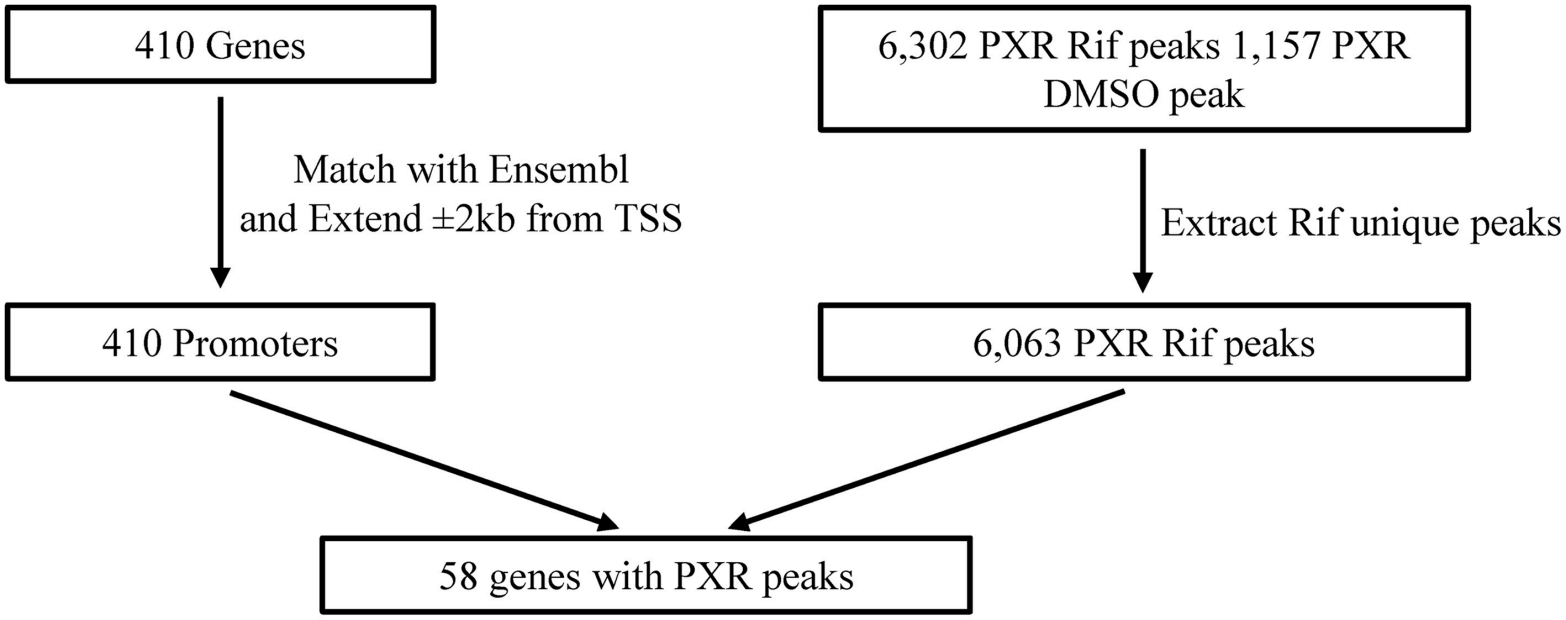
**Illustration of the ***in silico*** ChIP-Seq testing for PXR binding sites within the 410 transporters**. Rif, rifampin; TSS, Transcription Start Site.

**Table 4:** **The 58 transporters with ChIP-Seq determined PXR binding sites**.

**Gene**	**Transcript ID (Isoform)**	**Chromosome**	**Start of transcription start site (TSS)**	**Strand**	**Distance from the center of the PXR binding peak to the TSS**
*SLC2A1*	ENST00000415851	chr1	43424501	−	228.5
*SLC5A9*	ENST00000425816	chr1	48688357	+	−1875.5
*ABCD3*	ENST00000394233	chr1	94883933	+	−233
*SLC22A15*	ENST00000369503	chr1	116519119	+	884.5
*ANXA9*	ENST00000368947	chr1	150954493	+	97.5
*ATP1B1*	ENST00000367816	chr1	169074935	+	22.5
*SLC30A10*	ENST00000366926	chr1	220101945	−	2161.5
*ANXA11*	ENST00000481805	chr10	81928844	−	−190.5
*ABCC2*	ENST00000370449	chr10	101542489	+	−195
*SLC25A22*	ENST00000524891	chr11	795007	−	−46.5
*PEX16*	ENST00000527371	chr11	45937095	−	−2271.5
*SLC43A1*	ENST00000533515	chr11	57267151	−	2075
*SLC3A2*	ENST00000544377	chr11	62647894	+	470.5
*SLCO2B1*	ENST00000526839	chr11	74876919	+	−884
*CACNA2D4*	ENST00000588896	chr12	1905116	−	−248.5
*SCN8A*	ENST00000546961	chr12	51984050	+	424
*ATP2B1*	ENST00000428670	chr12	90102609	−	−682.5
*SLC41A2*	ENST00000432951	chr12	105352067	−	−234.5
*SCARB1*	ENST00000541205	chr12	125324218	−	1402
*ATP11A*	ENST00000487903	chr13	113344643	+	−604.5
*ABCA3*	ENST00000563623	chr16	2390599	−	−384.5
*SLC5A11*	ENST00000347898	chr16	24857162	+	−1215.5
*SLC7A5*	ENST00000563489	chr16	87870412	−	−1384.5
*SLC25A11*	ENST00000570543	chr17	4841853	−	−1323.5
*SLC47A1*	ENST00000571335	chr17	19436775	+	−62
*ABCC3*	ENST00000503304	chr17	48745791	+	−353.5
*ABCA9*	ENST00000461623	chr17	67045499	−	1577.5
*KCNJ2*	ENST00000535240	chr17	68164814	+	1032
*ATP5H*	ENST00000538432	chr17	73043046	−	190
*MFSD11*	ENST00000590070	chr17	74769179	+	2135
*SLC25A10*	ENST00000541223	chr17	79670404	+	87.5
*SLC16A3*	ENST00000582743	chr17	80186908	+	2067.5
*ATP9B*	ENST00000590608	chr18	76886267	+	−1304
*SLC25A42*	ENST00000318596	chr19	19174808	+	−476
*SLC1A4*	ENST00000493121	chr2	65215611	+	1472
*SLC20A1*	ENST00000272542	chr2	113403434	+	−488
*ATP5G3*	ENST00000392541	chr2	176046392	−	−105.5
*SLC23A2*	ENST00000423430	chr20	4880294	−	887.5
*SLC2A10*	ENST00000359271	chr20	45338126	+	124.5
*SLC5A3*	ENST00000381151	chr21	35445870	+	34.5
*PEX26*	ENST00000329627	chr22	18560689	+	−1844.5
*SLC25A1*	ENST00000461267	chr22	19165994	−	−31.5
*SCARB2*	ENST00000511129	chr4	77088018	−	949
*SLC39A8*	ENST00000394833	chr4	103266297	−	412
*SLC10A7*	ENST00000507030	chr4	147442870	−	−390
*SLC25A4*	ENST00000281456	chr4	186064395	+	15
*SLC26A2*	ENST00000286298	chr5	149340300	+	−390
*ANXA6*	ENST00000522664	chr5	150484834	−	319.5
*SLC39A7*	ENST00000444757	chr6	33168222	+	−418.5
*ABCC10*	ENST00000372515	chr6	43395104	+	618.5
*SLC29A1*	ENST00000393844	chr6	44187242	+	1915
*SLC17A5*	ENST00000393019	chr6	74363697	−	93.5
*SLC22A3*	ENST00000275300	chr6	160769300	+	1939.5
*ABCB4*	ENST00000453593	chr7	87104782	−	−315.5
*SLC37A3*	ENST00000340308	chr7	140098258	−	−58
*SLC20A2*	ENST00000518717	chr8	42396198	−	194.5
*ABCA2*	ENST00000492260	chr9	139922624	−	1220.5
*SLC25A5*	ENST00000317881	chrX	118602363	+	714.5

*In silico analysis utilizing a publically available HepG2 ChIP-Seq database to test the 410 transporter genes for PXR binding after rifampin treatment. Of the 410 transporter genes analyzed, 58 transporters were found to have PXR binding sites*.

## Discussion

Our study has several important first-time characterizations of rifampin's direct and indirect regulation of membrane transporters. First, rifampin regulates the gene expression of many members of the two major superfamilies (*ABC and SLC*) involved with clinically relevant drug transport. Second, the modulation of rifampin of transporter gene expression is associated with changes in miRNA expression. Third, we demonstrated that the effect of rifampin on transporter gene expression is tissue-specific. To the best of our knowledge, this study provides the first global characterization of rifampin induction of drug transporter gene expression.

Our studies demonstrate that rifampin treatment induces the gene expression of many members of the *SLCO, SLC*, and *ABC* transporter families. Twelve *ABC* transporter genes were regulated either directly or indirectly by rifampin; of these 12 *ABC* transporters genes, five of have previously been shown to be regulated by rifampin (Nishimura et al., 2002; Teng et al., 2003; Stapelbroek et al., 2010; Vilas-Boas et al., 2013; Weiss and Haefeli, 2013; Williamson et al., 2013) and two of the three *ABC* genes with the smallest *p*-values in our data set were drug transporters (*ABCC2, and ABCB1*). *ABCC2* and *ABCB1* have been shown to be induced by the nuclear receptor, PXR (Geick et al., 2001; Kast et al., 2002). Our analysis of a publically available ChIP-Seq dataset confirmed that *ABCC2* has a PXR binding site within the promoter region. *ABCB1* was not found to have a PXR binding site by our in silico ChIP-Seq analysis. PXR mediates at least part of the rifampin effects through direct binding to rifampin and nuclear localization (Stapelbroek et al., 2010; Honorat et al., 2011; Roques et al., 2013; Vilas-Boas et al., 2013; Weiss and Haefeli, 2013; Williamson et al., 2013). We likely did not identify the *ABCB1* PXR binding site within the promoter region due to defining the promoter region using the common convention of ± 2 kilobases around the TSS. In Kast et al. (2002), the *ABCB1* PXR binding site was over 7 kilobases from the TSS.

In the *SLC* family, the expression of 44 transporters were induced by rifampin. The most highly induced *SLC* transporter gene was the clinically relevant drug transporter *SLC51B* (*OSTbeta*; >12-fold), which is a newly recognized transporter that heterodimerizes with OST alpha and transports bile acids and conjugated steroids (Ballatori et al., 2013). For comparison, CYP3A4 was induced 22-fold (Ramamoorthy et al., 2013). We used a similar method of analyzing the data as Ramamoorthy et al., and with the same RNA-seq data set, CYP3A4 was induced 22-fold in our analysis as well. None of the *SLC* drug transporter genes (excluding *SLCOs*) have previously been shown to be induced by PXR or rifampin (Dixit et al., 2007). Our ChIP-Seq analysis identified that 33 *SLC* genes (Table 4 and Supplemental Table 7) had PXR binding sites. The two clinically relevant drug transporter *SLC* genes with a PXR binding peak identified in their promoter region were *SLC29A1*, and *SLC47A1*. The genes were induced by rifampin 1.38- and 1.37-fold (*p* < 0.05) respectively. *SLC51B* was not one of them. Although PXR and rifampin regulation of *SLC* transporters was mentioned in a review article (Ihunnah et al., 2011), we have been unable to find any published data supporting their statement for rifampin or PXR, but our ChIP-Seq analysis suggests that the rifampin induction of much of the *SLC* gene expression may be through other transcription factors (Rae et al., 2001; Hagenbuch and Meier, 2004).

In the *SLCO* family, two transporter genes (*SLCO4C1 and SLCO2B1*) were induced by rifampin; both are clinically relevant drug transporter genes. Neither of these *SLCO*s expressions have been previously shown to be altered by rifampin or PXR. Based on our ChIP-Seq data, *SLCO2B1* has a PXR binding site in their promoter.

Overall, five of the 12 rifampin induced clinically relevant transporters have PXR binding sites by ChIP-Seq analysis. With an additional sixth gene, *ABCB1*, having a published report of a PXR binding site, half of the rifampin induction of the clinical relevant transporter gene expression in this study was likely in part due to PXR activation. Our data suggest that a smaller portion of the induction of all *ABCs, SLCs, and SLCOs* gene expressions by rifampin is through PXR.

Furthermore, rifampin also reduced the gene expression of three clinically relevant drug transporters (*SLC15A1, SLC22A9, and SLCO1B3*). Of these transporter, only SLCO1B3 expression has studies evaluating rifampin regulation of gene expression. SLCO1B3 gene expression was shown not to change in primary hepatocytes when subjected to similar conditions (24 h 10 μM rifampin treatment; Williamson et al., 2013). Hafner et al. (2011) showed no change in SLCO1B3 after 48 h of rifampicin treatment. After 72 h of rifampin, SLCO1B3 is only moderately elevated (Dixit et al., 2007). These studies and ours suggest that the length of exposure to rifampin likely changes the level of SLCO1B3 gene expression. Additionally, Williamson et al. (2013) also showed an inhibition of SLCO1B3 expression as the rifampin concentration increased from 0.5 to 10 μM. A possible mechanism of this initial inhibition may be an indirect induction of repressors that reduce drug transport gene expression through PXR (Zhou et al., 2006; Tojima et al., 2012; Sehirli et al., 2015; Zhang et al., 2015). Also, we must consider that rifampin is activating other transcription factors. Another possibility may be inhibition of drug transport gene expression through induction, possibly through PXR of miRNAs (Ramamoorthy et al., 2013).

MicroRNAs are potentially involved in the effect rifampin exerts on the expression of clinically relevant drug transporter genes. For example, four transporters genes were negatively correlated with rifampin-induced miRNA expression: *SLC47A1/*miR-95*, SLC29A1/*miR-30d#, *SLC22A9/*miR-202*, and SLCO1B3/*miR-92a (Table 3). Only *SLCO1B3* and *SLC22A9* gene expression were reduced after rifampin treatment. This was surprising because no seed binding sites were predicted for the rifampin-induced miRNAs in the 3′ UTRs of these transporter genes (data not shown). Possibly, gene reduction may occur through less well understood miRNA binding in other mRNA regions (e.g., coding and 5′ UTR regions; Clark et al., 2014).

Rifampin's ability to alter gene expression patterns also demonstrated tissue specificity. Since the kidney proximal tubules express transporters that may contribute to plasma drug concentrations, we chose to study a proximal kidney cell line to explore the effects of rifampin on clinically relevant drug transporter gene expression. We used human proximal tubular epithelial cells, NHPTK, because of the known role of the proximal tubules in organic cation, anion, and drug transport (Giacomini et al., 2010; Kido et al., 2011). This cell line closely resembles primary proximal tubular cells with a renal phenotype that includes, parathyroid hormone responsiveness, tight junction formation, and transporter expression and function (Wieser et al., 2008; Herbert et al., 2013). High levels of drug transporter mRNA expression was detected, but rifampin did not induce or reduce transporter expression in a significant manner. This finding is in agreement with prior studies evaluating rifampin effects on individual renal drug transporters expression. For example, rifampin did not lead to alterations in *SLC22A2* expression in MDCK cells (Shu et al., 2001). Renal gene expression of *NR1I2* (*PXR)* is minimal in contrast to levels observed in liver and intestine (Cheng and Klaassen, 2006). Although renal transporter expression did not increase with rifampin treatment, this is likely secondary to low intracellular PXR protein concentrations. PXR appears to be a major determinant for rifampin's tissue selective regulation of drug transporters.

Our study has several strengths. First, primary human hepatocytes serve as an *in-vitro* system to predict changes in gene expression patterns in the human liver. Second, RNA sequencing allowed us to characterize all of the clinically relevant drug transporter genes that are expressed in human liver. Third, this approach allowed us to determine relationships between the gene expression patterns of the clinically relevant drug transporters, drug metabolizing enzymes, and miRNAs. Lastly, our findings provide a blue print for future investigations exploring rifampin's regulation of the transporter function.

There are a few secondary issues of some importance worth addressing. This study also produced a wealth of data on transporters that are not yet known to be clinically relevant for drug transport. In fact, the vast majority of *ABC* and *SLC* family members have not yet been shown to be clinically relevant to drug transport. The *SLC* superfamily is of particular interest in drug therapeutics as it includes transporters for e.g., serotonin, norepinephrine, and glucose that are drug targets (Gether et al., 2006; David-Silva et al., 2013; Felts et al., 2014). *SLC5A12* for instance is a sodium-coupled monocarboxylate transporter (SMCT-2) that regulates the lactic acid levels in the blood and has been linked to nicotine uptake (Ohkubo et al., 2012; Garnett et al., 2013); this gene was found in our study to be highly induced (>7-fold increase) by rifampin. Hence, our systematic characterization of transporter gene expression provides a more complete picture of the effects of rifampin on transporters at large. This information can inform the selection of gene targets for future mechanistic studies of transporter function.

Our study also has some limitations. Hepatocytes from only 7 different individuals is not sufficient to determine the population variability of the effects of rifampin. Using hepatocytes from seven donors for this study should identify the major transporters that are commonly regulated by rifampin (Kato et al., 2005); however, there may be some transporters that were not statistically significantly regulated by rifampin due to the low sample number, but are in fact regulated in some subpopulations of individuals. Another limitation is that the impact of genetic variation on expression levels was not taken into account. We also acknowledge that the fold-changes for the clinically relevant drug transporter gene expression were 1.18 to 1.85-fold. However, the levels of transporter mRNA expression have been highly correlated with their protein expression (when protein levels were analyzed by mass spectrometry; Ohtsuki et al., 2012; Lee et al., 2013). Also, these changes in expression levels represent a single time point, i.e., 24 h after rifampin treatment. Many of the correlations may not be direct, and represent inhibition of repressors or induction of co-activators. We also note that the ChIP-Seq dataset from HepG2 cells may not represent all of the PXR induction seen in primary hepatocytes, and limiting the promoter region to ± 2 kilobases around the TSS may have limited the detection of all PXR binding sites.

In conclusion we identified clinically relevant drug transporters exhibiting measurable changes that occur within a relatively short time period after treatment with rifampin. The coordinated changes of transporter expression by rifampin, and other potential inducing/repressing factors, may contribute to transporter-mediated drug-drug interactions. The rifampin-regulated transporters that we identified would be candidates for further investigations to understand the mechanisms (e.g., direct promoter regulation by PXR, indirect effects through miRNAs) involved in the up- and down-regulation of these transporters. Rifampin may serve as a representative ligand of many other PXR ligands (e.g., carbamazepine, St. John's Wort) may also cause effects on transporter expression similar to that of rifampin.

## Author contributions

Participated in research design: EB, TS, ME, ZD. Conducted experiments: EB, ME. Performed data analysis: EB, YL, HL, KB, MS, TS. Wrote or contributed to the writing of the manuscript: EB, ME, TS, ZD, AG, YL, KB, MS.

### Conflict of interest statement

The authors declare that the research was conducted in the absence of any commercial or financial relationships that could be construed as a potential conflict of interest.
