# Supplementary material for: Rifampin Regulation of Drug Transporters Gene Expression and the Association of MicroRNAs in Human Hepatocytes
Source: Front Pharmacol. 2016 Apr 26;7:111. doi: 10.3389/fphar.2016.00111 (PMC4845040; doi:10.3389/fphar.2016.00111)
Supplement: Supplementary file 1 [file Table1.DOCX]

**Supplemental Table 1: Primer sequences and annealing temperatures**

| Gene | F Primer | R Primer | Temp. (^o^C) |
| --- | --- | --- | --- |
| *GAPDH* | TGCACCACCAACTGCTTAGC | GGCATGGACTGTGGTCATGAG | 60 |
| *PXR^10^* | CTGGAGGTGAGACCCAAAGA | CACATACACGGCAGATTTGG | 58 |
| *SLC22A1^11^* | TGTGTAGACCCCCTGGCTA | GTGTAGCCAGCCATCCAGTT | 57 |
| *SLC22A5* | TTACCTTGGTGCCTACGAC | AAACAAGGTGAGGATGGCT | 54 |
| *SLC15A1* | CCGCCATCTACCATACGTTT | GAGCGACACAATGGTCTTGA | 58 |
| *SLC29A1* | ACCATTGGGATGTTTCCAG | AATGAAGTAACGTTCCCAGG | 55 |
| *SLCO4C1* | CTGCTATTGGCTATGTATTGGG | CAGTGACATCAGTGCTTTCTC | 54 |
| *MRP2* | ACCAATCCAAGCCTCTACCTAG | GAAAGTGCCACAGAGTATCGAG | 57 |
| *MRP4^12^* | GGACAAAGACAACTGGTGTGCC | AATGGTTAGCACGGTGCAGTGG | 62 |
