# Supplementary material for: Rifampin Regulation of Drug Transporters Gene Expression and the Association of MicroRNAs in Human Hepatocytes
Source: Front Pharmacol. 2016 Apr 26;7:111. doi: 10.3389/fphar.2016.00111 (PMC4845040; doi:10.3389/fphar.2016.00111)
Supplement: Supplementary file 6 [file Table6.DOCX]

**Supplemental Table 6: Differential expression of genes in NHPTK cells after rifampin treatment**

| Gene | Control | SEM | Rifampin | SEM | t-test *p*-value |
| --- | --- | --- | --- | --- | --- |
| *SLC22A1* | 1 | 0.257 | 1.210 | 0.274 | 0.492 |
| *SLC22A5* | 1 | 0.231 | 0.944 | 0.220 | 0.803 |
| *SLC15A1* | 1 | 0.227 | 1.035 | 0.336 | 0.906 |
| *SLC29A1* | 1 | 0.212 | 0.944 | 0.220 | 0.795 |
| *SLCO4C1* | 1 | 0.246 | 1.196 | 0.144 | 0.400 |
| *MRP2* | 1 | 0.291 | 1.366 | 0.250 | 0.285 |
| *MRP4* | 1 | 0.158 | 1.035 | 0.044 | 0.770 |
| *CYP3A4* | 1 | 0.088 | 1.776 | 0.444 | 0.117 |
