# Supplementary figures and images for: Rifampin Regulation of Drug Transporters Gene Expression and the Association of MicroRNAs in Human Hepatocytes
Source: Front Pharmacol. 2016 Apr 26;7:111. doi: 10.3389/fphar.2016.00111 (PMC4845040; doi:10.3389/fphar.2016.00111)

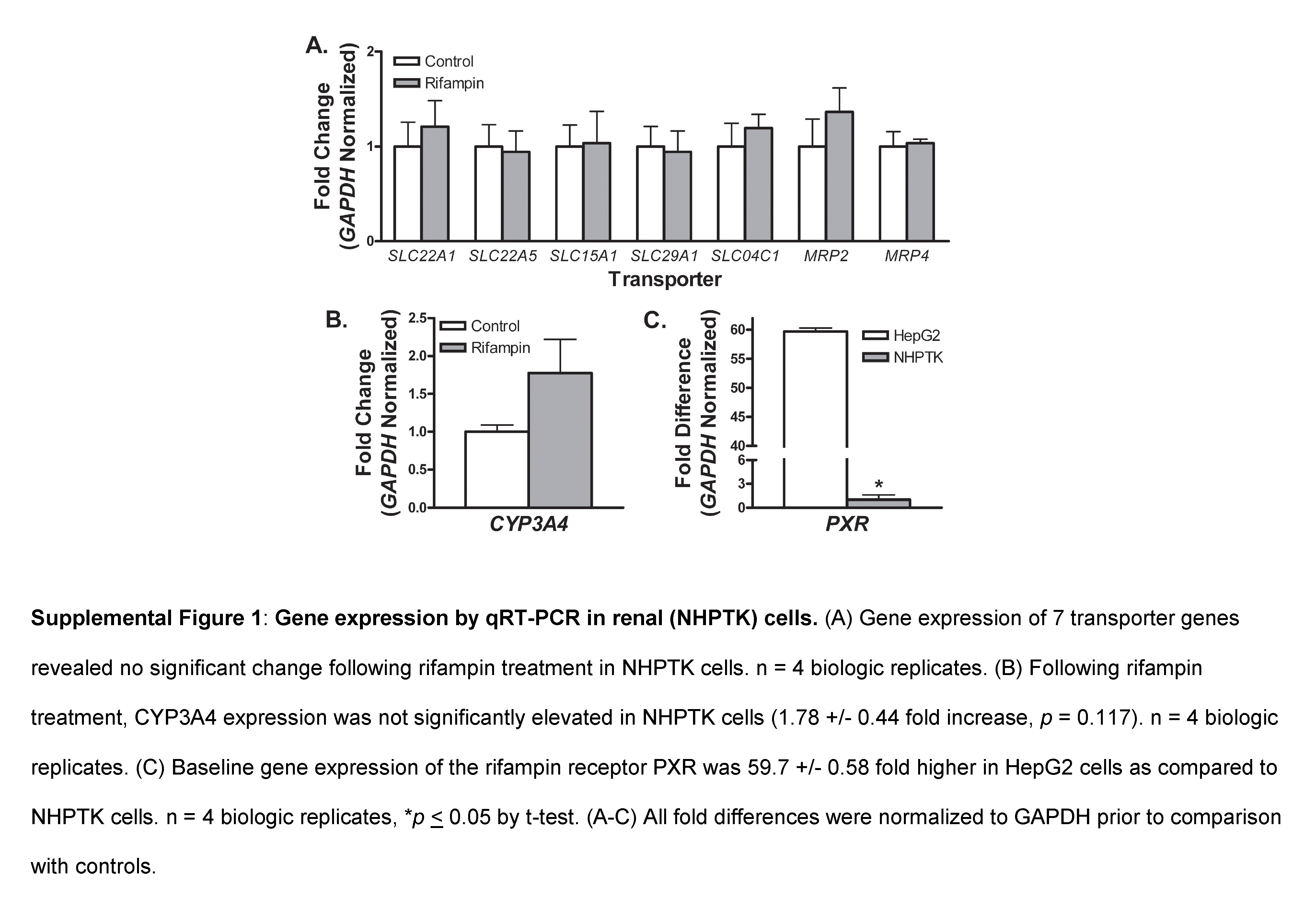

Supplement: Supplementary file 8 [file Image1.TIFF]
